# Pathways to seeking medication abortion care: A qualitative research in Uttar Pradesh, India

**DOI:** 10.1371/journal.pone.0216738

**Published:** 2019-05-13

**Authors:** Aradhana Srivastava, Malvika Saxena, Joanna Percher, Nadia Diamond-Smith

**Affiliations:** 1 Maternal and Child Health, Public Health Foundation of India, Gurgaon, Haryana, India; 2 Bixby Center for Population, Health, and Sustainability, University of California, Berkeley, CA, United States of America; 3 Department of Epidemiology and Biostatistics, Institute for Global Health Sciences, University of California, San Francisco, CA, United States of America; USC Keck School of Medicine, Institute for Global Health, UNITED STATES

## Abstract

**Introduction:**

Abortion is legal in India and medication abortion (MA) using a combined regimen of mifepristone and misoprostol is the preferred method. Users increasingly purchase MA kits directly from pharmacies, in some cases experiencing perceived complications and approaching a facility for care. We present findings of a qualitative research tracing the decision-making pathway(s) of MA users in Uttar Pradesh, India, to help understand knowledge and behaviour gaps, and recommend ways to improve the overall quality of care at these service delivery points.

**Methods:**

Forty in-depth interviews were conducted with recent MA users (20 each of clinic and pharmacy clients) across three districts. Providers were purposively selected in collaboration with an international organization selling MA kits, using their list of pharmacies and clinics. MA users were identified from the clients of the selected providers, and additionally through the snow ball method. Interviews were conducted in Hindi with verbal informed consent in a private place convenient to the respondent. Transcripts were translated to English and analysed thematically.

**Results:**

Users first sought MA kits at pharmacies out of convenience, low cost and customer anonymity. Men often purchased kits for their partners and trusted the chemist for guidance on dosage, progression and side effects. For side effects or other concerns after using an MA kit, users first visited their neighbourhood doctor or traditional practitioner. These providers either attempted to treat the issue and failed, or directly advised her to consult a gynaecologist. The final point of care was gynaecologists, preferably female private practitioners with their own clinics. They diagnosed most abortion-related cases as incomplete abortions, emptying the uterus using the dilation and curettage method. Comparatively low cost and convenience made users inclined towards repeat use of MA.

**Conclusion:**

There are information gaps at various stages in the MA pathway that need to be addressed. Large scale public information programmes are required on safe abortion care- when is it legal, where to obtain MA, dosage, side effects and signs of possible complications. Pharmacists could be trained or incentivized to improve their quality of care to facilitate adequate exchange of information on MA. Since, for most couples, the male partner purchases MA, information approaches or tools are needed that pharmacists can give men to share directly with the MA user.

## Introduction

Abortion has been legal in India for over four decades. The Government of India’s Medical Termination of Pregnancy Act, 1971, allows women to undergo abortion up to twenty weeks of gestation in a broad spectrum of conditions including contraceptive failure.[1] To expand availability, India’s National Population Policy envisaged the provision of abortion services in all public primary health facilities to individuals with pregnancies of up to eight weeks’ gestation.[2] Despite these policies, access to safe abortion services in facilities remains elusive for many in the country for barriers such as cost, lack of trained providers, physical accessibility of clinics, stigma, and misconceptions surrounding the legality of the service.[3] A 2015 study found that only 24% of primary health centres in Uttar Pradesh provide either abortion or post-abortion care, and most public facilities offering related services (64%) only offer post-abortion care.[4] Additionally, while the majority (74%) of women in Uttar Pradesh live in rural areas, only 39% of all facilities, private and public, which offer abortion-related services, are based in these areas.[4]

In 2002, the Drug Controller of India approved medication abortion (MA) using a combined regimen of mifepristone and misoprostol as an accepted abortion method.[5] This led to widespread and convenient availability of MA combination packs, or “kits,” in pharmacies across the country, even both drugs are under Schedule “H” in the country, not to be sold without prescription. In 2010, the Government of India further issued guidelines to train doctors in public health facilities on comprehensive abortion care, including both medication and surgical abortion. Though the government has taken progressive steps to increase access to legal and safe abortion services by amending policies in response to the changing abortion landscape in India, maternal mortality attributable to abortion-related complications remains high, accounting for an estimated 9% of maternal deaths in the country.[6] While many of the efforts to improve the quality of abortion services has focused on health facilities, data shows that individuals are increasingly purchasing MA kits directly from pharmacies, often without prescription. In addition to its widespread availability, the low cost of the medication compared to seeing private providers and the fact that MA, which studies suggest may often be preferred over surgical abortion, is frequently unavailable in public facilities, may be aiding the popularity of this practice.[7–9] In a recent study on abortion incidence, of the estimated 15.6 million abortions that took place in India in 2015, 81% (nearly 12.7 million) were estimated to have been done using MA. Almost three fourths (73%) of all abortions were through MA pills taken outside of facilities, possibly without medical advice or supervision.[10] Uttar Pradesh’s percentage is even higher; 83% of the estimated 3.15 million abortions that take place annually in the state are through out-of-facility MA use.[4] Due to gaps in pharmacist knowledge and little widely accessed information on the products, many individuals consume MA without complete knowledge on dosage, normal side effects, and possible complications.[11–15]

Uttar Pradesh (UP), India’s most populous state, performs poorly in most maternal and child health indicators, including low uptake of family planning and one of the highest maternal mortality ratios in the country. [16,17] According to the fourth National Family Health Survey (2015–16), the total unmet need for family planning in UP was high at 18.1%, with fewer than half (45.5%) of married women of reproductive age reporting current use of any family planning method, and less than a third (31.7%) using a modern method. [16] MA kits in UP, as in much of India, are widely available in pharmacies. Studies from other Indian states have reported that many pharmacists were providing MA kits readily without prescription and were either sharing inaccurate or no information while dispensing the MA kit to users. [11–15]

While studies exist that explore pharmacist knowledge on MA in India and their provision of the drugs, limited research, particularly qualitative research, explores pathways to out-of-facility MA use from the client’s perspective, despite its increasing popularity. A 2004 household survey on “Unwanted Pregnancy and Induced Abortion” in Rajasthan, surveyed women and men with a quantitative tool on their abortion experiences and knowledge and collected some information on abortion seeking pathways.[18] Twenty percent of women in the study who reported successful abortions said they had tried aborting on their own or went to an informal provider (n = 41). While two thirds of the women who attempted self-managed abortion had complete abortions, one third were unsuccessful, and ended up seeing an average of 1.1 formal providers to complete termination. A more recent study exploring the pathways to unsafe abortion in Madhya Pradesh, which employed both quantitative and qualitative methods, found that over half (53%) the women recruited in five government-run medical college hospitals and five district hospitals first attempted to abort at home, and 85% of those with “tablets.”[19] Additionally, 90% of the study participants who experienced post abortion complications first visited one or more local providers before seeking care at a district hospital or medical college, and 68% initially sought care from uncertified providers, like pharmacists. Another study in Bihar and Jharkhand found that 31% of women surveyed for eligibility for the study made at least one unsuccessful attempt to terminate a pregnancy by using medications or preparations at pharmacies before seeking care at a clinic.[20] While demographic characteristics of those who had attempted abortion before presenting to the health facility were examined, the study did not collect information on decision-making pathways or in-depth experiences with self-managed abortion.[20] Two other relevant studies in India examined women’s perspectives of and experiences with MA in clinics, but did not recruit women who had pursued out-of-facility abortion.[21,22] Qualitative studies with men on their experience as part of the out-of-facility MA decision-making process do not appear in the extant literature, despite evidence that they often purchase MA at pharmacies for their partners. [13–15]

In this context, qualitative research was conducted in UP, India, with the objective of tracing the decision-making pathway(s) of women and men seeking MA services. The goal of this study is to understand the gaps in knowledge and behaviour in depth, and the complexities of decision-making for the people who are actually purchasing MA and thus make recommendations for higher quality person-centred abortion care.

## Materials and methods

A total of 40 in-depth interviews (IDIs) were conducted with recent MA users in three districts in UP, India—the state capital Lucknow and two adjoining districts of Kanpur Nagar and Unnao. Twenty IDIs were conducted with clinic and 20 IDIs with pharmacy clients/users and/or their partners (both men and women). Of the total respondents, 20 were users and 20 were partners. Interviews were conducted by a team of experienced Indian qualitative researchers with a background in social science and public health.

The IDI guides for men and women who had purchased MA from a pharmacy or visited a clinician for MA covered the source of the user’s knowledge of MA, pathway of accessing it, nature of interaction with the provider and information received, experience after taking MA, barriers to obtaining care of desired quality and suggestions for information and support required for MA. All IDI guides were developed in English, translated to Hindi and then back-translated to check for consistency.

To recruit participants, we enrolled MA providers in collaboration with an international organization that manufactures and sells MA kits to pharmacists and clinics. The international organization shared a list of pharmacies and clinics who had purchased MA kits from them and who were likely to respond positively to the study. The shortlisted providers were then approached at their shops/clinics and requested to participate in interviews for another part of the study (not reported on here).

After the interview with the provider, they were requested to share contacts of clients for the interview of MA users, with the client’s permission. Clients who had used MA were contacted, asked to participate, and if interested, we set up a time for the interview. Interviews were conducted in a private place chosen by the respondent, and ranged from their home, the clinic itself, restaurants, cafes, malls or the survey team’s vehicle. A few interviews were also conducted telephonically. Verbal informed consent was obtained from the MA users before the interview. The interviews were conducted in Hindi and audiotape recorded. Once MA users were contacted and interviewed, further users to be interviewed were also identified through the snowball method by asking them to share contact details of any acquaintance of theirs who had also recently used MA services from a pharmacy or clinic. The interviews lasted for about 30–45 minutes. Ethical approval for this study was obtained from the University of California, San Francisco (153312) and the Institutional Ethics Committee of the Public Health Foundation of India (TRC-IEC-276/15). The study received ethics committee approval for obtaining verbal consent. Verbal consent was documented in a participant log maintained by the study team. Verbal informed consent was obtained from all participants for conducting and recording the interview.

IDIs were transcribed and translated to English. A team of four researchers analysed the data thematically using the Atlas.ti software. A thematic analysis approach to coding and interpreting the data was employed.[23] An initial list of codes was developed based on the interview guides and main aims of the study. After this a subset of interviews was coded by all team members, and the team met to discuss additional codes that emerged from the data to add and previously identified codes to adjust and expand as necessary. After coding another subset of interviews jointly, the team then discussed the codebook again, and finalized the codebook before proceeding with coding all interviews.

## Results

Over half of the respondents were first-time users of MA. Users’ age ranged from 20–30 years, while partners were aged between 22–34 years. All except one respondent (partner) were married. Parity ranged from 0–6, with three respondents having no children and about two-thirds having two or fewer children. Most were educated, with about three quarters having completed secondary school or above. All male partners were more educated than their female partners, while nine out of 20 users were illiterate or educated only up to the primary level. Only five respondents said that they were low income, while the rest were middle-income.

**Fig 1:**
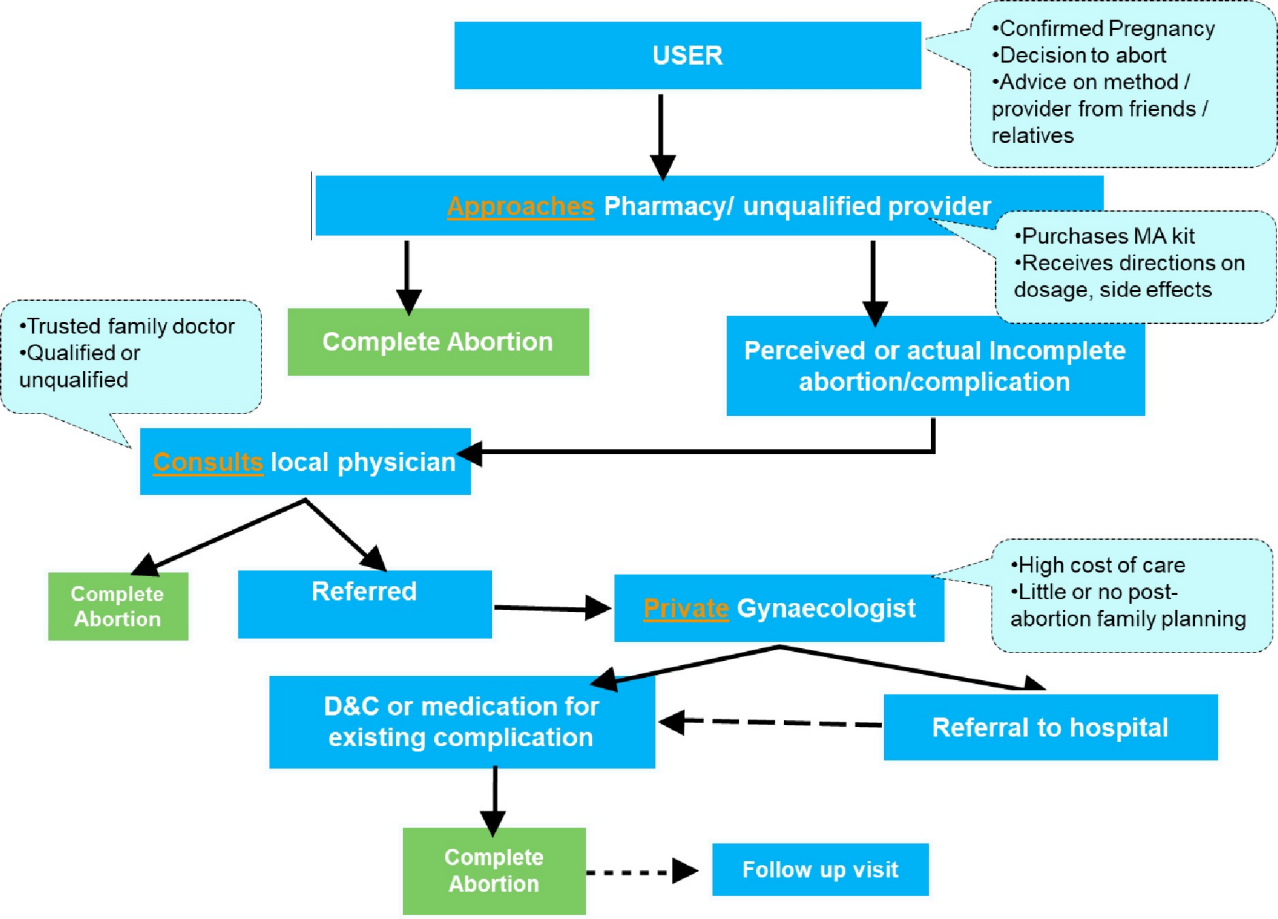
Pathway of seeking medication abortion care in Uttar Pradesh, India.

Based on the accounts of users’ experiences of MA, we have constructed a pathway of care seeking for MA, that appears strongly driven by gaps in user knowledge and awareness of MA, sometimes leading to incomplete or complications in abortions, multiple providers and time delays, and escalated user costs **(Fig 1)**.

### Pathways to care seeking: findings from qualitative in- depth interviews

#### Information on MA providers

Women decided on abortion either by themselves or jointly with their husbands/partners. A small number of women reported undergoing abortion under pressure from family members. Decision to seek abortion was followed by enquiry into the probable sources of abortion services. Women mostly approached their friends or relatives, while men spoke with their friends, relatives or colleagues, or known pharmacists to find out where to obtain an abortion.

*When my pregnancy was confirmed I knew I had to get abortion*. *I was considering visiting a doctor*, *so I asked my friend about it*. *She said I could do it myself by medication and did not need to visit a doctor*. *She said MA kits are easily available at medical stores and I don’t even need a prescription for it*. *So I went and bought MA kit from the medical store*. (Clinic client 16, Female, 23 yrs)

*I came to know about MA for the first time from my neighbor*. *She had taken it once*. *Recently one of my friends also used it*. (Clinic Client 9, Female, 30 yrs)

It is important to note that there was much confusion over the legal status of abortion, with some respondents thinking it was illegal, some not being sure, some knowing there are limitations, and others thinking that MA pills are legal, but surgical abortion not legal, or vice versa.

*I don’t have much information about it but I think it is illegal after a certain duration of pregnancy*. *I have read it in the newspaper that the court allowed a lady to abort the baby because of such and such reason*. *I think only in some special cases it is allowed that too with proper permission from the court*. (Pharmacy Client 14, Partner:Male, 29 yrs)

#### The first provider–Pharmacist or the neighbourhood unqualified provider (quack)

MA kits were sought at pharmacies in most cases, making pharmacies the first point of care. The convenience of MA availability, low cost, and the anonymity of the customer made pharmacies the preferred source of MA for most users. As in previous studies of out-of-facility MA use, usually men went to pharmacies to get the MA kit for their female partners.[14–16] For male purchasers, pharmacies were most often chosen based on trust and a previous relationship with the pharmacist.

*This chemist is one of my friends*. *Hence*, *I had already decided to consult and buy the medical abortion kit from him*. *I knew that he*, *being my friend*, *would advise me better than other chemists*. *He would be more reliable and accurate for buying the kit*. *He could also explain in detail about MA–he was the ultimate option for me*. *Therefore*, *I planned to buy medical abortion kit from this chemist*. (Pharmacy Client 2, Partner: Male, 32 yrs)

*This medical shop is near my house and we generally buy all other medicines from here*. *I know him well and trust him*. *I know he will give me good quality product and will keep my information confidential*, *that’s why I did not go anywhere else*. *I had the wrapper of my old MA kit*, *which I showed him and purchased the new pack*. *It cost me INR 350 (USD 5) for the new kit* (Pharmacy Client 16, Female, 29 yrs)

For women, and some men, the decision as to which shop to visit was based on confidentiality or concerns about stigma.

*I just randomly visited this chemist near my office and purchased the MA kit*. *Before getting into this medical store*, *I walked by two other stores too*, *but there were customers there*. *This one didn’t have any at that time*, *and the pharmacist was alone*. *So I thought it would be better as I could ensure my privacy there*. (Pharmacy Client 7, Partner:Male, 25 yrs)

*To get the MA kit I chose to visit a medical store which is far away from the place where I live–you know I did not want anyone to see me or recognize me*. *The one I went to is a big store and is located in the main market near the bus station*. *So many people come there–I was sure no one would recognize me and the chemist will also not remember me*. (Clinic client 11, Partner:Male, 29 yrs)

Customers usually explained their situation and asked for a medication to induce abortion. In most cases, users had little knowledge or preference for brands. They trusted the chemist to give them the appropriate medicine which would be effective and also advise them on dosage, normal progression, side effects and any other important information.

*I went to the chemist and told him that I confirmed my pregnancy through pregnancy test kit and now I want a medicine to abort this pregnancy*. *The chemist did not ask me any questions*, *just gave me four big tablets and four small tablets*. *He said they were from the MA kit*. *He charged me INR 400 (USD 5*.*7)*. *I don’t remember which brand the tablets were*. *He then explained about the dosage*. *He asked me to take two big tablets orally in the morning after breakfast with regular water*, *and two big tablets in the night after dinner*. *Then next day he said that I should take two small tablets orally with water in the morning after breakfast and remaining two small tablets at night after dinner*. *He said that by the second day heavy bleeding will take place along with pain in stomach and back*. *But this will happen only for 5–6 days and then the abortion will be complete*. *The chemist also told me to contact him if I face any kind of problem after taking the medicine*. (Clinic client 16, Female, 23 yrs)

*The chemist asked how many days had passed since the last menstrual cycle*. *I replied that about 28 days had passed since the last menstrual cycle*. *He said that the MA kit was safe to take within 90 days of pregnancy*. *He did not ask anything else about my wife*. *The chemist did not ask anything else about me—my name*, *whether I was married or how many children I had*, *nothing*. (Clinic client 18, Partner:Male, 25 yrs)

Sometimes local providers with no medical qualifications, commonly known as “quacks”, were consulted. Quacks offered relatively affordable care, including MA kits, and were often visited by women with low income and education levels

*When I found out that I was pregnant*, *I had no choice but to go for abortion*. *I shared my problem with my sister-in-law*. *I was thinking she would take me to a lady doctor*, *but she informed me that she had also undergone abortion recently using the MA kit*. *She asked me to try it*. *She said she had consulted a local doctor in the neighbourhood who had prescribed her the kit*. *She said I should also go there and get the kit from him*. (Clinic client 7, Female, 25 yrs)

Women had little or no prior knowledge of how to take the abortion medication and depended completely on the first provider–whether pharmacist or quack—for information on dosage, route of administration, normal progression, and possible signs of complication. In most cases this information was conveyed by their husbands or partners who had actually gone to get the medicine. The women took the medicine as advised by the chemist or unqualified private providers and had a complete abortion in several cases. However, if there was a complication or they were unsure or scared about the side effects they were experiencing, they then visited a second provider.

#### Additional follow up–treatment of MA-related concerns

Some women experienced what they perceived to be complications after taking the MA kit or had side effects that worried them. Prolonged bleeding was the most common issue. Respondents also reported experiencing commonly documented side effects expected with MA, such as severe stomach pain, cramps, dizziness and nausea, vomiting, extreme weakness and fever. Some women faced difficulty in seeking additional care when they had not informed their family about the abortion and feared that they would come to know. Sometimes they delayed treatment thinking that the issue would resolve on its own.

*Both of us did not want a baby*. *My husband also thought why to go and consult a doctor; they will charge more*. *I confirmed my pregnancy through pregnancy test kit at home and then decided to take MA kit*. *It didn’t cost much too—my husband bought it for 320 rupees (USD 4*.*6) from the chemist*. *However*, *this time the bleeding did not stop and continued for more than 20 days*. *In fact I started feeling very weak*, *and couldn’t even perform household chores properly*. *My children started asking*, *“What is wrong with you mummy*?*” That’s when I decided to see the doctor and came to this clinic*. (Clinic Client 9, Female, 30 yrs)

Side effects, most commonly prolonged bleeding, led to worry among women, as many of them had not been adequately or correctly informed about them. It is possible that information was lost in transit from pharmacist to male partner who purchased the kit, and further to the female partner who consumed it as per her partner’s instructions. It is also possible that the pharmacists or health workers themselves gave erroneous information on dosage and possible, anticipated side effects versus signs of complications. Regardless of reason, additional cost of treatment for the side effect was a stressful, often unanticipated financial burden on the users.

*As I said*, *we didn’t know anything about MA*. *… And we also didn’t know who could give us the right information or treatment*. *My wife thought the ASHA (community health worker) would know everything*, *but her information was wrong*. *So she ended up with this prolonged bleeding and we had to eventually come to this doctor*. *Overall I have spent INR 7000 (USD 100) now for my wife’s treatment* … *This is a financial crisis for us* … *Moreover*, *my wife is facing so much difficulty in coping with the complications alone… I think we might have avoided these all problems if my wife had not taken MA tablets by her own*. *If only we had known*, *we would have directly come to the doctor*. *But what to do*, *it is in the fate of the poor to suffer always*. (Clinic Client 19, Partner:Male, 30 yrs)

*The chemist did not say anything about the side effects of MA*. *He only gave me the MA kit and said it would successfully terminate my wife’s pregnancy*. *He explained the dosage and how to take the medicine and then he said that there would be bleeding for 4 to 5 days and nothing more about it*. *Now I wish he had given us more information*. *Some of the abdominal pain was not as worrying as somehow my wife expected it*, *but the prolonged bleeding was scary*. *We wondered what had happened*, *whether the abortion was complete or not*, *whether some other condition had developed*. (Clinic client 18, Partner:Male, 25 yrs)

Women generally delayed seeking care to help with management of side effects. When they did seek care, they tended to visit their neighbourhood traditional providers or quacks. Essentially the second provider was the familiar “physician” often consulted by the family, who could be qualified or unqualified, practising modern or traditional medicine. Sometimes they were also providers referred by friends or relatives. These providers either attempted to treat the woman themselves, or directly advised them to consult a gynaecologist. In the second situation, women and partners again consulted their friends or relatives about another provider who could treat the issue.

*Actually*, *we first consulted an Ayurvedic doctor in our neighbourhood*. *We generally consult him for small ailments*. *He asked the complications in detail and then suggested that we consult a gynaecologist*. *He also said that we should not delay in visiting a gynaecologist otherwise my wife’s complication might turn out to be severe*. *Then we decided to get treatment from this doctor as this doctor is known to us for a long time*. *She is one of my distant relatives* too. (Clinic client 6, Partner:Male, 25 yrs)

*I came because my bleeding wouldn’t stop*. *It continued for 20 days*. *I felt weak and was really scared*. *I told my neighbour about my condition and she suggested that I consult this doctor*. *She had consulted her previously*. *She said the doctor was very good*. (Clinic client 16, Female, 23 yrs)

#### Gynaecologists–the final point of care

Sometimes, when women could not get relief from the treatment provided by the second provider, they would approach a third provider. By this time the provider was almost always a trained gynaecologist, as women believed they would be successfully treated by that provider, after the second provider failed to resolve their issue successfully, or directly advised them for specialist care. Preferred gynaecologists were female private practitioners practising in their own private clinics. Most abortion related cases reaching gynaecologists were treated as incomplete abortions. Generally, gynaecologists emptied the uterus of retained matter through the dilatation and curettage (D&C) procedure.

*The doctor asked if my wife had completed the MA course*. *My wife replied no*, *as she had not taken all the MA tablets*. *The doctor asked her why she did not take all the tablets*. *My wife replied that the bleeding had begun after taking the second medication*, *so she did not continue the tablets further*. *She said that the ASHA*, *from whom the MA kit was purchased*, *had also said that she could discontinue the tablets once the bleeding started*. *The doctor then asked us whether we had consulted any other doctor before reaching her*. *I replied that we did not consult any doctor*. *She asked if I had come with money or not*. *I asked how much I had to arrange for*. *The doctor said at least INR 5000 (USD 71*.*4) would be needed*. *We then went back*. *I arranged for the money*, *then came back to her clinic after three days*, *and deposited the money to the doctor*. *The doctor prescribed for ultrasound and blood test*. *The ultrasound and blood test report came on the same day*. *The doctor told my wife to come with an empty stomach the next day*, *as she had to undergo D&C*. (Clinic client 19, Partner:Male, 30 yrs)

Clients reported preferring to buy MA kits from pharmacies over consulting a gynaecologist in the first place on account of the huge cost difference–the MA kit costs between INR 350–500, (USD 5–7) while the consultation with the gynaecologist costs women INR 1500–5000 (USD 21–71), depending on the procedure performed.

*Yes*, *if there is a need for abortion*, *I would prefer to taking MA rather than seeking institutional abortion*, *as I think it is quite safer and convenient to use at low cost*. (Pharmacy client 1, Partner:Male, 30 yrs)

*I used the MA kit two times and both times I was worried about whether it would harm my health in any manner*, *but I didn’t know whom to ask about all this*. *Consulting a doctor would have cost me money*, *so I just prayed and took the MA*. (Pharmacy Client 16, Female, 29 yrs)

Such experiences by women of possible incomplete abortions and subsequent D&C procedures, whether medically necessary or not, influence users’ decisions on future abortion care seeking. Generally, women who reported having a negative experience with MA stated that they would not repeat MA and if by any chance they had to have another abortion, they would visit a clinician directly.

*I initially thought that it was convenient and safe to take MA*. *I did not ever think that there could be complications in taking MA and we may have to consult a gynaecologist*. *Now I feel one should not take MA without consulting a doctor*. *Our doctor was also telling us this*. *She explained that while it is true that taking MA looks very convenient but it is not yet fully safe*. *If I had consulted a doctor before giving MA kit to my wife*, *she would not have had to undergo D&C today*. *Now I feel that D&C should be preferred over taking MA*, *as taking MA is more risky than undergoing D&C*. *Yes*, *it is true that D&C is much more expensive than MA*. *Therefore I think it is important to arrange an amount of INR 3000 to 4000 (USD 43–57) if one decides to go for abortion*. (Clinic client 6, Partner:Male, 25 yrs)

A fourth of the respondents expressed that they would consider using MA in the future too, if the need arose. As expected, women who were able to have a complete abortion using the MA kit and did not suffer any significant side effects were positively inclined to it and believed they would use it again if needed.

*Yes*. *We prefer the [MA] kit only; it is more trustworthy and we would visit the same store for purchasing the kit in future* (Pharmacy client 3, Partner:Male, 31 yrs)

However, women who had a bad experience in terms of experiencing a side effect and then going for further treatment, involving a high cost, said that they would not use MA again in future or not use it without consulting a doctor. Some women said they would not consume MA pills again as they worried it would impact their future fertility.

*Formerly I thought that MA was convenient and safe to use but now*, *I am not very sure*. *Any wrong dosage can have very serious consequences*. *I suffered a lot when I was bleeding–so much weakness*! *And all that blood loss was scary*! *Fortunately*, *I underwent my abortion safely but it was really a horrible experience for me this time*. *I now believe that one should not take MA without consultation of a doctor* (Clinic Client 17, Female)

*The chemist was not very helpful*, *I must say*. *After this bitter experience I will never prefer to take MA kit in future*. (Clinic Client 5, Female, 36 yrs)

## Discussion

Our findings suggest a general lack of awareness among users about the source, dosage, progression, and side effects of MA. This led to a dependence on the first provider they approached–in most cases pharmacists—for all information. In cases where the pharmacist provided incomplete or inaccurate information, women were at risk of taking the medications incorrectly and having little knowledge about what to expect. This reflects other studies in India that found a link between poor pharmacist knowledge and poor client knowledge. A recent study of pharmacists selling MA in Delhi found that only around 40 percent were aware of the correct dosage and administration of the mifepristone and misoprostol combination. [14] Most pharmacists were not aware of any side effects or complications and were also indifferent to the needs of clients. Most pharmacists did not ask the client’s gestational age, confirmation of pregnancy, or explain side effects or failure of MA. None of them asked any obstetric history or gave contraceptive advice. [14]

The lack of knowledge about MA creates a situation where women do not know when they are experiencing complications compared to normal side effects, or that complications could require specialized care. In a state-level study of Uttar Pradesh, researchers found that about half of individuals seeking care for abortion-related complications presented with an incomplete abortion after self-administered MA use.[4] Many of these cases, as well as those with prolonged or abnormal bleeding, likely represented a normal progression of MA and would not have needed an intervention had users been given more information on the process. Twenty four percent of all women attempting an abortion in Uttar Pradesh in 2015, about 1.22 million, received treatment for complications in a facility, a complication treatment rate higher than other South Asian countries with data on the indicator, aside from Pakistan.[4] A study conducted in the states of Gujarat and Jharkhand in India by Boler et al in 2009 found 66% of women purchasing MA directly from pharmacies reported perceived complications following their abortion [15] This study also found that poor quality information from the initial provider may lead women towards more invasive, often unnecessary procedures, such as D&C.

These findings suggest that there are information gaps at various stages in the MA pathway that need to be addressed in a user-friendly and confidential manner. There is a high dependence on informal networks (friends, relatives or colleagues) for information at all levels (what to do in case of unwanted pregnancy, whom to first approach for MA, whom to approach if there is an issue following MA kit use). While informal networks and seeking information from friends and family is common in healthcare, there is a need to have more easily accessible, correct information on a product that millions of women in Uttar Pradesh, and over ten million women in India, access each year.

This study did not find as much stigma around using MA as anticipated. Some users (especially women) sometimes sought services from a provider who was less crowded, so others could not hear, or farther from their home, so people that they knew would not see them, due to stigma about seeking abortion. However, overall, there was less stigma, which may be a result of all clients having used MA and thus having low enough stigma to be willing to seek the product.

Recommendations include the need to launch large-scale public information programmes on safe abortion care, including the gestational age in which MA kits, intended to be used up to nine weeks, are effective, where to obtain MA, the recommended regimen, possible side effects, and where to go for the treatment of complications. Furthermore, many users believed that abortion was illegal in India or were confused about the status, echoing findings in other studies.[15,18,24] This accentuates the secrecy and stigma around the process, inhibiting users from seeking timely and quality care for complications. Therefore, educating the public that abortion is legal could help reduce the stigma surrounding seeking MA amongst users. Particularly, clear distinction has to be made in the messages between MA within nine weeks of pregnancy, which is legal, and sex-determination, which is illegal in India [25].

Though gender inequality and role of women in decision-making are not the focus of this paper, the study found that a few female clients did report pressure from husbands to abort, and gender inequality in decision-making, especially about using family planning, did play into some women’s decision-making processes. Women’s low status in India generally, and lack of ability to seek health care alone or without chaperons or male support in many cases, likely limit women’s access to the MA, abortion generally, or other reproductive health options that they desire.[26,27]

As pharmacists often are the first point of care and the main source of abortion medication and guidance, they should be trained to inform customers adequately to ensure clients know what to expect when taking MA. Since potential clients seek care from providers they know, and are less likely to want to return to the same provider when issues following medication use arise, pharmacists have a natural incentive to improve their quality of care to facilitate adequate exchange of information on MA. Recent evidence from Nepal shows that training pharmacy workers led to safe and effective MA sale to almost all clients. [28] Furthermore, since for most couples the male partner is the first point of contact at the time of MA purchase, it is essential to develop information approaches or tools that can enable pharmacists to provide information to those people ultimately using the medication. In contrast to other studies that examined decision-making around abortion in India, women rarely reported undergoing abortion under pressure from their husband / partner, and therefore partners have more potential to be a source of support and information. [29] It is possible that written or pictorial information pamphlets that are provided with MA, or finding ways to engage men to ensure that they pass along accurate information to their partners, could be effective methods. A hotline for women self-managing their abortion, a model employed in other settings around the world, could be another tool directly linking MA users with support and information. The hotline could be promoted at the pharmacies through signs, on the hand-outs, or even directly on the MA kit.

This study suffers from the limitations of generalizability common to qualitative research studies. It cannot be generalized to represent the national context in India. Also, being concentrated in urban and peri-urban areas, it does not effectively capture the MA user pathway in rural areas. Despite these limitations, we upheld practices in maintaining key tenants of validity in qualitative data collection and analysis, as outlined by Maxwell (1992), including generalizability and, descriptive, interpretive and theoretical validity.[30] The multi-country nature of our team, including local Indians familiar with the context and who participate in components of data collection/supervision, helped us be able to ethically and credibly collect and contextualize our data.

### Conclusions

Our in-depth qualitative findings add to the literature by describing the pathway of seeking medication abortion and subsequent care and information for women and men in urban India. Given the growing magnitude of use of MA, especially outside of facilities, understanding when, where and why people seek care and information is important for knowing the key points for intervention and support. Mapping the pathways taken by women seeking abortion care elucidates the information gaps that affect a safe and satisfactory abortion experience. There is a strong need to educate providers and clients on the legality of abortion, proper administration of MA drugs, normal side effects, and when and where to seek treatment should a complication arise. Such interventions can significantly bring down the burden of unsafe abortions and improve the overall experience of abortion seekers in and out of facility.

## Supporting information

S1 File In-depth interview guide. (PDF)

## Abbreviations

MA: Medication abortion
UP: Uttar Pradesh
D&C: Dilation and curettage
