# Supplementary material for: Pathways to seeking medication abortion care: A qualitative research in Uttar Pradesh, India
Source: PLoS One. 2019 May 13;14(5):e0216738. doi: 10.1371/journal.pone.0216738 (PMC6513085; doi:10.1371/journal.pone.0216738)
Supplement: S1 File — (PDF) [file pone.0216738.s001.pdf]

Interviews with clients after receiving MA

1. Have you heard about MA before? Where? क्या आप ने सुना है की गोलिया लेने से भी गर्भपात हो सकता है? कहाँ से सुना है?

a. Do you know someone who has taken it? क्या आप ऐसे किसी को जानते है जिसने अभी हाल ही मे गोली ली हो?

2. Have you ever taken MA before? क्या आप ने कभी पहले गर्भपात की गोली ली है?

a. How many times? कितनी बार ली है?

b. Can you tell me about that/those experience(s)? When? What happened? क्या आप मुझे उस अनुभव के बारे मे कुछ बताएँगी? गोली कब ली था और क्या क्या हुआ था विस्तार से बताए?

3. Why did you decide to seek services from this provider/chemist? आप ने इसी स्वस्थ्य सेवा प्रदाता/ दुकान वाले से सेवा (गोली) लेने का निर्णय क्यो लिया था ?

i. Pathway: did you go somewhere else first? क्या आप गर्भपात कराने के लिए यहाँ आने से पहले किसी और के पास भी गयी थी ?

ii. Where, why, what happened कहाँ गयी थी /कब गयी थी / वहाँ क्या हुआ था?

b. Why this provider/chemist and not other? क्यो इसी स्वस्थ्य सेवा प्रदाता/ दुकान वाले को आपने चुना किसी और को क्यो नहीं चुना?

c. Have you had previous experience with this provider/chemist? क्या इसी स्वस्थ्य सेवा प्रदाता/ दुकान वाले से आप ने कभी पहले भी गर्भपात कराया है या गोली ली है

d. Did you hear about this provider/chemist from someone else? क्या आप ने इस सेवा प्रदाता/ दुकान वाले के बारे मे पहले भी कभी किसी से सुना था ?

e. Probe on Convenience, price, आप को इस सेवा प्रदाता/ दुकान वाले से गर्भपात की गोली लेने मे सुविधा या आसानी लगती है? आप को यह सेवा लेने मे कुल कितने पैसे खर्च करने पड़े थे?

4. How long did you wait to come here after finding out you were pregnant? आप को जब पता चला की आप गर्भवती है उसके बाद से कितने समय और इंतजार करने के बाद आप इस सेवा प्रदाता/ दुकान वाले से गर्भपात की गोली लेने के लिए आई?

5. Can you describe the interaction with the provider/chemist today? क्या मुझे आज इस सेवा प्रदाता/ दुकान वाले से आप ने गर्भपात की गोली कैसे ली इस विषय मे विस्तार से बताएँगी?

a. What questions did the provider/chemist ask (health, LMP) प्रदाता/ दुकान वाले ने आप से क्या सवाल पूछे (स्वास्थ्य और आखिरी माहवारी की तारीख के बारे मे)

b. Did the provider/chemist ask you questions about marital status, age, parity, etc. क्या सेवा प्रदाता/ दुकान वाले ने आपसे आपके वैवाहिक स्थिति, आयु, बच्चो की संख्या आदि के बारे मे सवाल पूछे थे?

c. Did you feel that you had privacy during your visit? Can you tell me how or how not? क्या सेवा प्रदाता/ दुकान वाले से आप ने एकांत/ अकेले मे बात किया था ? कृपया मुझे बताए की आप ऐसा कैसे कर सकी या नहीं कर पायी ?

i. Is privacy important to you when seeking MA services? क्या आप के लिए गर्भपात की गोली लेने के समय एकांत होना जरूरी है?

d. Did you feel that you had confidentiality during your visit? Can you tell me how or how not? क्या सेवा प्रदाता/ दुकान वाले के यहा गोली लेते हुये आप गोपनीयता महसूस करती है (आप को यकीन है की वो आप की बात को गोपनीय रखेगा)? कृपया मुझे बताए की आप ऐसा कैसे कर सकी या नहीं कर पायी.

i. Is confidentiality important to you when seeking MA services? क्या आप के लिए गर्भपात की गोली लेने मे गोपनीयता होना जरूरी है?

e. Did you feel that you were treated with respect during your visit? Can you tell me how or how not? क्या सेवा प्रदाता/ दुकान वाले ने आप से आदर / सम्मान के साथ बात की थी? कृपया मुझे बताए की आपको ऐसा कैसे लगता है या नहीं लगता है?

i. Is respect important to you when seeking MA services? क्या आप के लिए गर्भपात की गोली लेते समय आदर / सम्मान से बात करना जरूरी है?

f. Did you feel that you trusted your provider? Can you tell me how or how not? क्या आप सेवा प्रदाता/ दुकान वाले पर भरोसा रखती है ? कृपया मुझे बताए की आप ऐसा कैसे कह सकती है या भरोसा नहीं करती है ?

i. Is trust important to you when seeking MA services? क्या आप के लिए गर्भपात की गोली लेते समय सेवा प्रदाता/ दुकान वाले पर भरोसा रखना जरूरी है?

6. Can you tell me what the provider/chemist told you about MA? क्या आप मुझे बताएँगी की गर्भपात की गोली देते समय इस सेवा प्रदाता/ दुकान वाले ने आपको इसके बारे मे क्या क्या बताया?

i. What did the provider/chemist tell you about how to take the medication? गर्भपात की गोली देते समय सेवा प्रदाता/ दुकान वाले ने क्या आपको बताया की ये गोली कैसे खाना है?

ii. Did the provider/chemist tell you to take the first medication? Can you describe how to me? क्या सेवा प्रदाता/ दुकान वाले ने पहली गोली कैसे लेनी है इस संबंध मे आपको कुछ बताया था? क्या आप मुझे बता सकती है की पहली गोली कैसे लेनी है?

iii. Did the provider/chemist tell you to take the second medication? Can you describe how to me? क्या सेवा प्रदाता/ दुकान वाले ने दूसरी गोली कैसे लेनी है इस संबंध मे आपको कुछ बताया था? क्या आप मुझे बता सकती है की ये गोली कैसे लेनी है?

iv. How do you feel about inserting the medicine vaginally? क्या गर्भपात की गोली को योनि के अंदर रखने मे आप सहज महसूस करती है या आपको कैसा लगता है?

1. Did the provider/chemist explain how to do this? क्या सेवा प्रदाता/ दुकान वाले ने आपको बताया था की यह कैसे करना है?

2. Have you ever done this before? क्या आपने ऐसा पहले भी किया है?

v. Do you know if you have other options for taking the second medicine? क्या आप को पता है कि दूसरी गोली लेने के और भी तरीके है?

1. Can you describe that to me? क्या आप मुझे बता सकती है की दूसरी गोली लेने के और कौन से तरीके है?

b. What did the provider/chemist tell you about what to expect during and after taking MA? क्या सेवा प्रदाता/ दुकान वाले ने आपको बताया था कि गोली लेने के समय और उसके बाद क्या क्या संभावित परिणाम/असर होंगे?

c. What did the provider/chemist tell you about what side effects are normal and what are not? क्या सेवा प्रदाता/ दुकान वाले ने आपको बताया था कि गोली लेने से कौन से संभावित सामान्य असर होंगे और कौन से सामान्य बुरे/ खतरनाक असर होंगे?

d. What did the provider/chemist tell you about when to seek care and where to go? क्या सेवा प्रदाता/ दुकान वाले ने आपको बताया था कि गोली लेने के बाद किस समय / परिस्थिति मे आपको स्वास्थ्य जांच के लिए जाना जरूरी है और आपको कहाँ जाना चाहिए?

e. Did you take first dose already? क्या आप पहली गोली अभी ले चुकी है?

i. Can you show me the pill packet that they gave you? क्या आप मुझे वो दवा का पत्ता दिखा सकती है जो आप को सेवा प्रदाता/ दुकान वाले ने दिया था?

f. Are you going back to provider/chemist? When? क्या आप सेवा प्रदाता/ दुकान वाले के पास दुबारा जाएंगी? कब जाएंगी?

7. Did the provider/chemist give you any additional information (written, visual) क्या सेवा प्रदाता/ दुकान वाले ने आप को कोई भी और सहायक जानकारी दी थी (लिखित या छपी हुई)

a. Was there any other information that you would have liked during your interaction with the provider/chemist? आपके और सेवा प्रदाता/ दुकान वाले से क्या कोई और भी अन्य जानकारी आप चाहती थी?

i. What would be the best way of getting that information? Leaflet, website, links to website, आप के अनुसार ये जानकारी प्राप्त करने का सबसे अच्छा साधन कौन सा है? छपा हुआ पर्चा, वेबसाइट, वेबसाइट के लिंक?

8. Did anyone come to the facility with you to get MA? क्या आप के साथ सेवा प्रदाता/ दुकान वाले के यहा गर्भपात की गोली के लिए कोई और भी आया था?

a. Who? Did you feel that they provided you support? कौन आया था ? क्या आप को लगता है उसने आपको मदद /सपोर्ट किया?

b. If not, would you like someone to have come with you? Who? अगर ऐसा नहीं है तो आपको लगता है कि किसी को आप के साथ आना चाहिए था आप के सपोर्ट के लिए ? वो कौन हो सकता है ?

i. Family, health worker आपके घर का या स्वास्थ्य कार्यकर्ता?

c. Is there anyone at home who knows that you will be doing a MA, क्या घर मे कोई और है जिसे पता है कि आप गर्भपात के लिए गोली लेने जा रही है?

i. Will this person provide care for you at home? क्या यह व्यक्ति घर मे आपकी देखभाल करता है ?

ii. If not, would you want someone to care for you at home? Who? यदि ऐसा नहीं है तो क्या आप चाहती है की कोई घर मे कोई आपकी देखभाल करे? वो कौन हो सकता है ?

9. What are the main things important to you for receiving MA services? गोलियों द्वारा गर्भपात'की सेवा लेने के लिए, आप के लिए सबसे जरूरी बातें क्या हैं ?

10. What are the main barriers to receiving the care you want? जो सुविधा/देखभाल आप चाहती हैं उसको लेने के लिए आपके सामने सबसे बड़ी रुकावट /अवरोध क्या है?

a. What could be done to reduce these barriers? इस रुकावट /अवरोध को कम करने के लिए आपके हिसाब से क्या किया जा सकता है?

11. Did you feel treated differently by the provider/chemist due to any personal characteristics? क्या आप को लगता है आपकि किन्ही व्यक्तिगत विशेषताओ के कारण सेवा प्रदाता/ दुकान वाले ने आपके साथ कुछ अलग तरीके से व्यवहार किया?

a. Probe: age, marital status, parity, religion, caste, etc.) (आयु, वैवाहिक स्थिति, बच्चो की संख्या, धर्म, जाती आदि)

12. Did the provider/chemist talk to you about family planning after your MA? क्या सेवा प्रदाता/ दुकान वाले ने आप को गर्भपात की गोली देने के बाद आपसे परिवार नियोजन के बारे मे बात की?

a. Did you purchase a method at this time? क्या आप ने परिवार नियोजन का कोई भी साधन इस बार खरीदा है?

13. How much did you pay for the MA today? आप ने गर्भपात की गोली के लिए आज कितना पैसा दिया है /कितने मे खरीदी है?

14. Is abortion legal in India? क्या गर्भपात भारत मे वैधानिक (कानूनी तौर पर मान्यता प्राप्त) है?

a. Are there any cases where it is not legal? क्या ऐसे कोई केसेस है जिसमे यह वैधानिक नहीं है?

Follow up

1. Did you complete the abortion (all pills)? क्या आप का गर्भपात हो गया है ? (सारी गोलिया खा ली है?

a. How did you feel about the vaginal insertion? गर्भपात के लिए गोली को योनि के अंदर रखना आपको कैसा लगा था?

2. Did you have any side effects or complications? क्या आपको कोई साइड इफैक्ट या परेशानी हुयी थी?

a. Please describe कृपया विष्तार से बताए

3. Did you go to a health facility because of these side effects or complications? क्या आप किसी स्वास्थ्य सुविधा केंद्र पर इन साइड इफैक्ट या परेशानियों के कारण गयी थी?

4. Did you feel that you had enough information from your /chemist about how to do the MA?
क्या आपको लगता है की आप को आपके सेवा प्रदाता/ दुकान वाले ने गर्भपात की गोली से गर्भपात करवाने के बारे मे पर्याप्त जानकारी दी थी ?

5. What additional information would you have liked? आप क्या अतिरिक्त जानकारी चाहती थीं ?

a. Would you have liked a leaflet that you could take home with instructions? क्या आप कोई छपा हुआ पर्चा या किताब चाहती थीं जिसमे सभी निर्देश दिये गए हों और जिसे आप घर ले जा सकें?

b. Website you could go to? Phone number to contact? कोई वेबसाइट जिसपर आप को सारी जानकारी मिल सके? कोई फोन नंबर जिस पर आप बात करके जानकारी प्राप्त कर सके?

6. Would you use MA again? क्या आप दुबारा कभी गर्भपात के लिए गोलियों का इस्तेमाल करेंगी?

7. Would you go back to the same provider/chemist for MA or other services again? क्या आप उसी सेवा प्रदाता/ दुकान वाले से दुबारा गर्भपात की गोली लेना अथवा अन्य सुविधाए लेना पसंद करेंगी जिससे आप ने इस बार गोली ली थी?
